# The Sale of Arsenical Beer

**Published:** 1901-02-02

**Authors:** 


### THE SALE OF ARSENICAL BEER.

The question of the liability of the beer seller who serves his customers with arsenical beer will evidently have to come before the higher courts, as the Manchester magistrates have given diametrically opposite decisions on the subject. By Section 3 of the Sale of Food and Drugs Act it is enacted that "no person shall mix . . . any article of food with any ingredient or material so as to render the article injurious to health." But in Section 5 it is provided that no liability is incurred if the accused person can show that he was unaware of the admixture and could not with reasonable diligence have ascertained it. Section 6, however, prohibits the sale "to the prejudice of the purchaser," of any article of food, including drink, "which is not of the nature, substance, and quality of the article demanded," whether the seller knows of its condition or not. At first sight this would seem to cover everything, and Mr. Headlam at the Manchester City Police Court is reported to have taken it as so doing. It would, he said, be an extraordinary thing that a person who unknowingly sold milk with water in it should be liable to a penalty, and that a person who sold beer with arsenic in it should be allowed to go free. So he convicted. Certainly the analogy is striking, but analogy is not always a safe guide. Mr. Yates, at the County Police Court, however, held that there had been no offence. He held that the whole of the Act must be read together, and that, seeing that in Section 3 the words used are 'injurious to health," while in Section 6 they are "to the prejudice of the purchaser," and that in regard to these two sections both the penalties and the defences are different, the legislature must have meant to deal with different things in these two sections—in the one with something injurious to health, and in the other with something prejudicial to the purchaser in some other way. The particular case, then, came under Section 3, in regard to which Section 5 held the seller blameless. He therefore dismissed the summons. Both magistrates granted cases for a superior court.

